# Identification and characterization of genes that control fat deposition in chickens

**DOI:** 10.1186/2049-1891-4-43

**Published:** 2013-11-09

**Authors:** Hirwa Claire D’Andre, Wallace Paul, Xu Shen, Xinzheng Jia, Rong Zhang, Liang Sun, Xiquan Zhang

**Affiliations:** 1 Rwanda Agriculture Board, Research Department, P. O Box 5016, Kigali, Rwanda; 2 Council for Scientific and Industrial Research (CSIR), Animal Research Institute, P. O. Box AH 20, Accra, Achimota, Ghana; 3 Department of Animal Genetics, Breeding and Reproduction, College of Animal Science, South China Agricultural University, Guangzhou, Guangdong 510642, China

**Keywords:** Chicken, Fat deposition, Genes

## Abstract

**Background:**

Fat deposits in chickens contribute significantly to meat quality attributes such as juiciness, flavor, taste and other organoleptic properties. The quantity of fat deposited increases faster and earlier in the fast-growing chickens than in slow-growing chickens. In this study, Affymetrix Genechip® Chicken Genome Arrays 32773 transcripts were used to compare gene expression profiles in liver and hypothalamus tissues of fast-growing and slow-growing chicken at 8 wk of age. Real-time RT-PCR was used to validate the differential expression of genes selected from the microarray analysis. The mRNA expression of the genes was further examined in fat tissues. The association of single nucleotide polymorphisms of four lipid-related genes with fat traits was examined in a F_2_ resource population.

**Results:**

Four hundred genes in the liver tissues and 220 genes hypothalamus tissues, respectively, were identified to be differentially expressed in fast-growing chickens and slow-growing chickens. Expression levels of genes for lipid metabolism (*SULT1B1*, *ACSBG2*, *PNPLA3*, *LPL*, *AOAH*) carbohydrate metabolism (*MGAT4B*, *XYLB*, *GBE1*, *PGM1*, *HKDC1)*cholesttrol biosynthesis (*FDPS*, *LSS*, *HMGCR*, *NSDHL*, *DHCR24*, *IDI1*, *ME1*) *HSD17B7* and other reaction or processes (*CYP1A4*, *CYP1A1*, *AKR1B1*, *CYP4V2*, *DDO*) were higher in the fast-growing White Recessive Rock chickens than in the slow-growing Xinghua chickens. On the other hand, expression levels of genes associated with multicellular organism development, immune response, DNA integration, melanin biosynthetic process, muscle organ development and oxidation-reduction (*FRZB*, *DMD*, *FUT8*, *CYP2C45*, *DHRSX*, and *CYP2C18*) and with glycol-metabolism (*GCNT2*, *ELOVL 6*, and *FASN*), were higher in the XH chickens than in the fast-growing chickens. RT-PCR validated high expression levels of nine out of 12 genes in fat tissues. The G1257069A and T1247123C of the *ACSBG2* gene were significantly associated with abdominal fat weight. The G4928024A of the *FASN* gene were significantly associated with fat bandwidth, and abdominal fat percentage. The C4930169T of the *FASN* gene was associated with abdominal fat weight while the A59539099G of the *ELOVL 6* was significantly associated with subcutaneous fat. The A8378815G of the *DDT* was associated with fat band width.

**Conclusion:**

The differences in fat deposition were reflected with differential gene expressions in fast and slow growing chickens.

## Background

Fat deposition is a crucial aspect in modern chicken breeding schemes because it is associated with selection for increased body weight in broilers [1-7]. The growth of broiler chicken is accompanied by an increased percentage of body fat with a concomitant increase in the mass of abdominal and visceral fat [8]. The quantity of fat deposited increases faster and earlier in fast-growing chickens than in slow-growing chickens [9-12]. Excessive adiposity is a problem in modern broiler industry [13]; and needs to be controlled to reduce negative effects on productivity, acceptability, and health of consumers. In meat-type chickens, excessive adipose tissue decreases both feed efficiency during rearing and the yield of lean meat after processing. However, fat is the major contributor to meat flavor; and the presence of intramuscular fat confers high eating quality of meat. Therefore, regulating fat deposition plays an important role in broiler chicken production.

In birds, lipogenesis, takes place primarily in the liver whereas adipocyte serves as the storage site for triglycerides [14]. Hepatic lipogenesis contributes 80 to 85% of the fatty acids stored in adipose tissue [15] because lipogenic activity in chickens is much greater in the liver than in adipose tissue [16-18].

In the past decade, genetic mechanisms underlying chicken fat deposition were widely studied but few studies were conducted to determine the gene expression involved in pathways as well as mechanisms that lead to adiposity in chickens [19]. In the present study, fast-growing White Recessive Rock chickens (WRR) and slow-growing Xinghua chickens (XH) were used to characterize specific genes for fat deposition in chickens. Global gene expression patterns within the liver and hypothalamus tissue of WRR and XH chickens were determined using Partek GS 6.4 Affymetrix Genechip® Chicken Genome Arrays and the differentially expressed genes were identified. Some of the differentially expressed genes were validated by determining their mRNA expression in liver, hypothalamus and fat tissues. The association of single nucleotide polymorphisms of the genes with chicken fat traits was also investigated.

## Materials and methods

### Chicken populations

Eight WRR (4♂ + 4♀, Institute of Animal Science, Guangdong Academy of Agricultural Sciences, Guangzhou, China), and 8 XH chickens (4♂ + 4♀, Fengkai Zhicheng Poultry Breeding Company, Guangdong, China), were used for differential expression observation with microarray hybridization. All the birds were fed a nutritionally balanced corn-soybean diet [20]. The birds had free access to water. They were slaughtered at 8 wk of age, and the liver and hypothalamus were excised, snapped frozen in liquid nitrogen and stored at -80°C until required for further analyses.

Six sets of WRR (3♂ + 3♀), and another six of XH (3♂ + 3♀), were used to study mRNA expression of the *SULT1B1*, *PNPLA3*, *GPAM*, *ELOVL6*, *LPL*, *FASN*, *ACSBG2*, *FDPS*, and *FRZB* genes in abdominal fat, subcutaneous fat, breast muscle, and pituitary tissues in the liver and hypothalamus tissues.

For association analysis, an F_2_ resource population was constructed by crossing WRR with XH chickens [21]. The fat traits such as abdominal fat weight, subcutaneous fat thickness, fat band width, abdominal fat percentage were recorded in all F_2_ full-sib individuals.

### Ethics statement

The study was approved by the Animal Care Committee of South China Agricultural University (Guangzhou, People's Republic of China). Animals involved in this study were humanely sacrificed as necessary to ameliorate their suffering.

### Microarray hybridization and data preprocessing

Total RNA was isolated from frozen tissues (50 mg) using TRIzol reagent (Invitrogen, CA, USA) according to the manufacturer’s instructions. Total RNA concentration was determined by spectrophotometry. The RNA labelling and microarray hybridization were carried out according to the Affymetrix Expression Analysis Technical Manual (Biochip Corporation, Shanghai, China). The arrays were scanned using the Affymetrix Scanner 3000.

The GeneChip Chicken Genome Array used in the present study was created by Affymetrix Inc. (Santa Clara, USA) at the end of 2006, with comprehensive coverage of over 38,000 probe sets representing 32,773 transcripts corresponding to over 28,000 chicken genes (Chicken Genome Sequencing Consortium 2.1). Sequence information for this array was selected from the following public data sources: GenBank, UniGene and Ensembl.

Data normalization was used to eliminate dye-related artifacts. Consecutive filtering procedures were performed to normalize the data, and to remove noise derived from absent genes, background, and nonspecific hybridizations. Comparisons of expression levels were performed for each gene, and genes with the most significant differential expression (*P* < 0.05) were retained. Raw data sets were normalized to total fluorescence, which represents the total amount of RNA hybridized to a microarray, using the Partek GS 6.4 (Affymetrix Genechip® Chicken Genome Arrays, USA). QVALUE was used to obtain false-discovery rates (FDR).

The data obtained were subjected to Partek GS 6.4 for comparison using Affymetrix Expression Console Software, for expression algorithm robust multi-array (RMA) analysis. Multivariate ANOVA was used to determine significant differences among the replicates. Differentially expressed genes between WRR and XH chickens were identified by cutoff of fold-change (fold change) ≥ 2 and *P* < 0.05. Molecular functions of differentially expressed genes were classified according to molecule annotation system (MAS) 3.0 (http://bioinfo.capitalbio.com/mas3/). Database from the Kyoto Encyclopedia of Genes and Genomes (KEGG) were used for pathway analysis on differentially expressed genes using AgriGO (GO Analysis Toolkit and Database for Agricultural Community) http://bioinfo.cau.edu.cn/agriGO/) and Database for Annotation, Visualization and Integrated Discovery (DAVID) Bioinformatics Resources (http://david.abcc.ncifcrf.gov/).

### Validation of the differential expression with real-time RT-PCR

The primers were designed based on the published cDNA sequences of *SULT1B1*, the *LPL*, *ELOVL6*, *ACSBG2*, *SCD5*, *FADS1*, *PNPLA3*, *GAPDH*, *BEAN*, *SLC22A2*, *DDT*, *PLA2G12A*, and *18S* genes (http://www.ncbi.nlm.nih.gov) using GENETOOL software (BioTools, Alberta, Canada). The RNA was reverse-transcribed using the RevertAid Fist Strand cDNA Synthesis (Toyobo, Japan). After reverse transcription, the cDNA of the selected genes were amplified by real-time reverse transcription PCR. The relative level of each mRNA normalized to the *18 s* gene was calculated using the following equation: fold change = 2^Ct target (WRR)–Ct target (XH)^/2^Ct *18S* (WRR) -Ct 18S (XH)^. The linear amount of target molecules relative to the calibrator was calculated by 2^- ΔΔCT^. Therefore, all gene transcription results are reported as the *n*-fold difference relative to the calibrator. Specificity of the amplification product was verified by electrophoresis on a 0.8% agarose-gel. The results were expressed as mean ± SE.

### Fat tissue expression of the differential expression genes with real-time RT-PCR studies

The same primers as those used in validation were used for determining fat tissue expression. The real-time RT-PCR reactions were performed using the iCycler Real-Time PCR detection System. Each sample reaction was ran in triplicate and the expression quantified as the number of cycles (CT) after which fluorescence exceeds the background threshold minus the CT for the housekeeping control (ΔCT). The calculation of absolute mRNA levels was based on the PCR efficiency and the threshold cycle (Ct) deviation of unknown cDNA versus the control cDNA. The quantitative values were obtained from the Ct values, which were the inverse ratios relative to the starting PCR product. The linear amount of target molecules relative to the calibrator was calculated by 2^- ΔΔCT^. Briefly, the relative levels of each mRNA were expressed as the same as above.

### SNP identification and association analysis

Tree variation sites were identified in intronic of chicken genes *ACSBG2*, *FASN and ELOVL6;* and one variation site was identified as non synonymous of chicken *ACSBG2 and* synonymous coding region of chicken *DDT* gene by using GENBANK (Table 1).

**Table 1:** The identified SNPs of the 4 fat deposition related genes

**Variation ID**	**Genes name**	**Chr.**	**Position on chromosome (bp)**	**Consequence to transcript**	**Allele**
rs10731268	*ACSBG2*	28	1257069	NON_SYNONYMOUS_CODING	G/A
rs15248801	*ACSBG2*	28	1247123	INTRONIC	T/C
rs15822158	*FASN*	18	4928024	INTRONIC	G/A
rs15822181	*FASN*	18	4930169	INTRONIC	C/T
rs15822181	*ELOVL6*	4	59539099	INTRONIC	A/G
rs14092745	*DDT*	15	8378815	SYNONYMOUS_CODING	A/G

SNP position was determined based on the reported SNP in ensembl http://www.ensembl.org/biomart/martview.

*ACSBG2*, acyl-CoA synthetase bubblegum family member 2; *FASN*, fatty acid synthase; *ELOVL6*, elongation of long chain fatty acids; *DDT*, D-dopachrome tautomerase.

The data for association study were analyzed by ANOVA (SAS 8.1). The statistical significance threshold was set at *P* < 0.05. Values were expressed as the mean ± SEM, and the differences in the means were compared using Duncan’s Multiple Range Test at 5% level of significance.

## Results

### Differentially expressed genes in fast-growing WRR and slow-growing XH chickens at 8 wk of age

**Figure 1:**
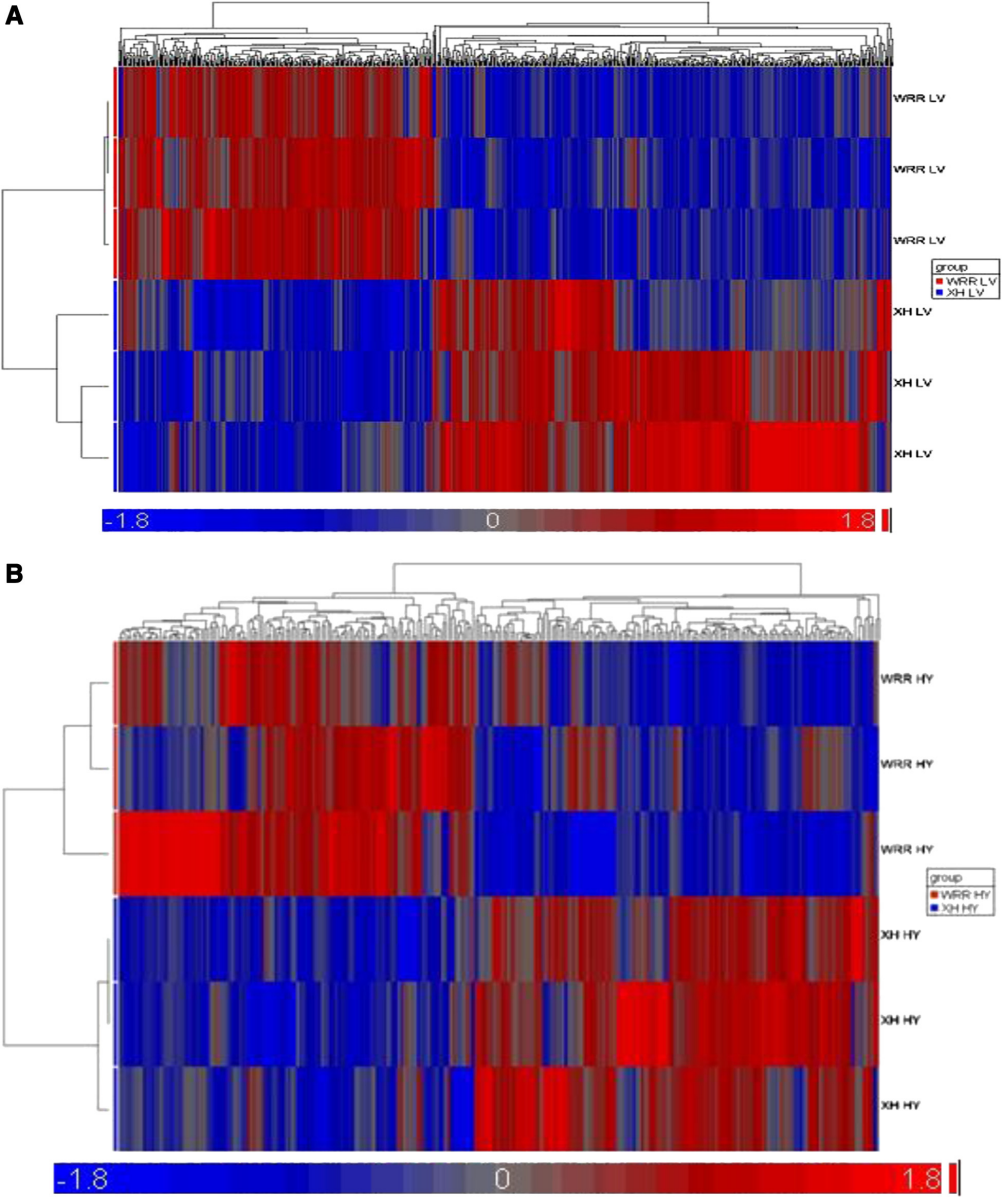
**Heat maps of differentially expressed genes of FG and SG chicken during developmental stages of liver and hypothalamus tissue. A** Heat map of differentially expressed genes of FG and SG chicken during developmental stages of liver tissue. The red color represents fast growing chicken (WRR) genes while the blue color represents the slow growing chicken (XH) genes. The fold changes were arranged from -1.8 up to 1.8 (*P* < 0.05). WRRLV means liver tissue from White Recessive chickens. XHLV means liver tissue from Xinghua chickens. **B** Heat map of differentially expressed genes of FG and SG chicken during developmental stages of Hypothalamus Tissue . The red color represents fast growing chicken (WRR) genes while the blue color represents the slow growing chicken (XH) genes. The all identified gene, fold changes were arranged from -1.8 up to 1.8 (*P* < 0.05). WRRHY means Hypothalamus tissue from White Recessive chickens. XHHY means Hypothalamus tissue from Xinghua chickens.

After normalization and statistical analyses, 400 and 220 genes with at least 2-fold differences were identified (*P* < 0.05, FC ≥ 2) in liver and hypothalamus tissues of WRR and XH chickens, respectively. When fast-growing WRR chickens were compared with slow-growing XH chickens, 214 and 91 genes were up-regulated, and 186 and 129 genes were down-regulated in liver and hypothalamus tissues (Figure 1A and B; Tables 2 and 3).

**Table 2:** Fold-changes of significantly differential expressed genes in WRR and XH chickens

**Gene symbol**	**Gene title**	***P* value**	**Fold change**	**Chromosome alignment s**
**Lipid metabolic process**				
*SULT1B1*	sulfotransferase family, cytosolic, 1B, member 1	0.0001	7.689	chr4:53309684-53311980
*ACSBG2*	acyl-CoA synthetase bubblegum family member 2	0.004	5.382	chr28:1247898-1259038
*LPL*	lipoprotein lipase	0.018	2.528	chrZ:53399697-53408327
*AACS*	acetoacetyl-CoA synthetase	0.021	2.507	chr15:4477440-4512637
*PNPLA3*	Patatin-like phospholipase domain containing 3	0.024	3.028	chr1:71256654-71270462
*AOAH*	acyloxyacyl hydrolase (neutrophil)	0.043	-2.516	chr2:46723433-46778195
**Carbohydrate metabolic process**				
*MGAT4B*	mannosyl (alpha-1,3-)-glycoprotein beta-1,4-N-acetylglucosaminyltransferase, iso	8.33E-05	2.178	chr13:13578206-13590970
*XYLB*	xylulokinase homolog (H. influenzae)	0.0001	2.603	chr2:6032066-6115406
*GBE1*	glucan (1,4-alpha-), branching enzyme 1 (glycogen branching enzyme, Andersen dis	0.0008	2.119	chr1:98522850-98669948
*PGM1*	phosphoglucomutase 1	0.002	2.179	chr8:28644700-28665874
*HKDC1*	hexokinase domain containing 1	0.038	7.368	chr6:11960338-11966483
**Fatty acid biosynthetic process**				
*ELOVL6*	ELOVL family member 6, elongation of long chain fatty acids	0.002	2.181	chr4:59493262-59560594
*FASN*	fatty acid synthase	0.029	2.840	chr18:4906222-4942593
**Cholesterol biosynthetic process**				
*LSS*	lanosterol synthase (2,3-oxidosqualene-lanosterol cyclase)	0,001	2,186	chr7:6878402-6888484
*HMGCR*	3-hydroxy-3-methylglutaryl-Coenzyme A reductase	0,005	3,236	chrZ:23472632-23474241
*FDPS*	farnesyl diphosphate synthase (farnesyl pyrophosphate synthetase, dimethylallylt	0,021	2,167	chrUn_random:7545445-7546725
*DHCR24*	24-dehydrocholesterol reductase	0,026	2,587	chr8:26011324-26019531
*HMGCR*	3-hydroxy-3-methylglutaryl-Coenzyme A reductase	0,027	2,805	chrZ:23472597-23491333
**Oxidation reduction**				
*CYP1A4*	cytochrome P450 1A4	0,001	9,342	chr10:1822784-1826314
*CYP1A1*	cytochrome P450, family 1, subfamily A, polypeptide 1	0,003	6,485	chr10:1806680-1809495
*DHRSX*	dehydrogenase/reductase (SDR family) X-linked	0,004	-2,1001	chr1:132739051-132944192
*LOC418170*	similar to aldose reductase	0,014	2,042	chr1:64269892-64273020
*CYP2C45*	cytochrome P-450 2C45	0,019	-5,673	chr6:17648418-17654233
*AKR1B1*	aldo-keto reductase family 1, member B1 (aldose reductase)	0,028	2,788	chr1:64293981-64312331
*MICAL1*	microtubule associated monoxygenase, calponin and LIM domain containing 1	0,029	-2,186	chr26:25422-27136
*CYP2C18*	cytochrome P450, family 2, subfamily C, polypeptide 18	0,040	-3,214	chr6:18655324-18664396
*CYP4V2*	cytochrome P450, family 4, subfamily V, polypeptide 2	0,048	2,426	chr4:63195381-63202122
*DDO*	D-aspartate oxidase	0,049	2,219	chr3:69194822-69198140
**Cicardian clock genes**				
*ARNTL*	aryl hydrocarbon receptor nuclear translocator-like	0,002	-2,043	chr5:8501344-8546127
**Transforming growth factor beta receptor signaling pathway**				
*FUT8*	fucosyltransferase 8 (alpha (1,6) fucosyltransferase)	0,028	-2,473	chr5:24711230-24725772
**Asparagine biosynthetic process**				
*ASNS*	asparagine synthetase	1,48E-05	9,945	chr2:24628018-24641745
**Melanin biosynthetic process**				
*DDT*	D-dopachrome tautomerase	3,50E-05	-13,908	chr15:8372896-8375331

Positive values indicated that the genes were up-regulated when fast growing WRR chickens are compared with slow growing XH chickens.

Negative values meant down-regulation when comparison between WRR and XH chickens are made, Data were significantly different (*P* > 0,05), and fold changes were not smaller than 2.

“---” meant unknown.

**Table 3:** Differentially expressed genes in hypothalamus of WRR and XH chickens

**Gene symbol**	**Gene title**	***P*** **value**	**Fold-change**	**Chromosomes alignment**
**Lipid metabolic process**				
*P20K*	quiescence-specific protein	0,0109	-2,402	chr17:881078-883996
**Porphyrin biosynthetic process**				
*FECH*	ferrochelatase (protoporphyria)	0,009	-2,155	chrZ:267090-278253
**Nitrogen compound metabolic process**				
*RCJMB04_35g11*	vanin 1	0,0406	-2,312	chr3:58745711-58758866
**Transport**				
*PMP2*	peripheral myelin protein 2	0,031	3,405	chr2:126148069-126152515
**Ion transport**				
*SLC22A2*	solute carrier family 22 (organic cation transporter), member 2	0,004	-2,123	chr3:47342914-47355320
*GLRA1*	glycine receptor, alpha 1 (startle disease/hyperekplexia)	0,035	-2,146	chr13:12903161-12940509
**sodium ion transport**				
*SLC13A5*	solute carrier family 13 (sodium-dependent citrate transporter), member 5	0,003	-2,046	chr19:9754843-9769124
**Copper ion transport**				
*SLC31A1*	solute carrier family 31 (copper transporters), member 1	0,017	7,335	chr17:1874758-1884555
**Striated muscle contraction**				
*MYBPC2*	myosin binding protein C, fast type	0,034	-3,717	un
**Response to stress**				
*HSP70*	heat shock protein 70	0,001	-2,093	chr5:55409752-55412248
*HSP25*	heat shock protein 25	0,006	-2,759	chr27:4486260-4487251
**Signal transduction**				
*LOC419724*	similar to KIAA0712 protein	0,009	-2,393	chr24:1172171-1264246
**cyclic nucleotide catabolic process**				
*N4BP2L1*	NEDD4 binding protein 2-like 1	0,006	3,732	chr1:178835487-178837064
**Negative regulation of endothelial cell proliferation**				
*TNFSF15*	tumor necrosis factor (ligand) superfamily, member 15	0,027	2,553	chr17:2943951-2959613
**Regulation of transcription, DNA-dependent**				
*CREB3L2*	cAMP responsive element binding protein 3-like 2	0,001	-2,197	chr1:59488049-59500378
*MLL3*	myeloid/lymphoid or mixed-lineage leukemia 3	0,008	-4,110	chr2:6484781-6486027
**Protein complex assembly**				
*ATPAF1*	ATP synthase mitochondrial F1 complex assembly factor 1	0,003	-3,241	chr8:22570693-22574048
**Protein amino acid phosphorylation**				
*PRKD3*	protein kinase D3	0,0005	-2,745	chr3:34793675-34819927
*SGK1*	serum/glucocorticoid regulated kinase 1	0,004	2,419	chr3:58130872-58134430
*RIPK2*	receptor-interacting serine-threonine kinase 2	0,013	2,532	chr2:129010265-129029220
**Proteolysis**				
*FOLH1*	folate hydrolase (prostate-specific membrane antigen) 1	0,005	-2,344	chr1:191872775-191933212
*C1R*	complement component 1, r subcomponent	0,011	-2,014	chr1:80553474-80558910
*ITGBL1*	integrin, beta-like 1 (with EGF-like repeat domains)	0,024	2,43	chr1:147579457-147711379
*VPS13B*	vacuolar protein sorting 13 homolog B (yeast)	0,041	-2,155	chr2:133389475-133390863

Positive values simply mean that the genes were up-regulated when WRR chickens are compared with XH chickens.

Similarly, negative values mean down-regulation when comparison between WRR and XH chickens is made, Data were significantly different [*P* > 0, 05 (fold change ≥ 2)].

In the liver, lipid metabolism genes viz *SULT1B1*, *ACSBG2*, *LPL*, *AACS*, *PNPLA3*, were up-regulated while *AOAH* gene was down-regulated. The carbohydrate metabolism genes: *MGAT4B*, *XYLB*, *GBE1*, *PGM1*, and *HKDC1*, were up-regulated (Table 2; Figure 1A). The fatty acid biosynthesis genes, *ELOVL6* and *FASN*, cholesterol biosynthesis genes, *LSS*, *HMGCR*, *FDPS*, *DHCR24*, malate metabolism process gene, *ME1*, proline biosynthesis process genes, *PYCR2* and *ALDH18A1*, oxidation-reduction reactions genes, *CYP1A4*, *CYP1A1 similar to aldose reductase*, *AKR1B1*, *CYP4V2*, and *DDO*, cyclic nucleotide catabolic process gene, *N4BP2L1*, and multicellular organism development genes, *SEMA5A* and *C1orf107*, were identified highly expressed in WRR chickens. In contrast, genes highly expressed in XH chickens were associated with multicellular development, *FRZB*, immune response, DNA integration, melanin biosynthetic process, *DDT*, muscle organ development, *DMD*, transforming growth factor beta receptor signaling pathway, *FUT8*, and oxidation-reduction, *CYP2C45*, *DHRSX*, *MICAL1*, and *CYP2C18*. In addition, the genes for the biosynthesis of steroids and fatty acid, *ELOVL6*, and *FASN* were also observed highly expressed in XH chickens (Table 2; Figure 1). The metabolic process genes, *ACSM5* (hypothetical protein), were down- regulated by 5- fold, while another metabolic process genes, *ENPEP*, were up-regulated by 5- fold (Table 2).

In the hypothalamus, the cyclic nucleotide catabolism gene, *N4BP2L1*, was up-regulated in fast growing WRR chickens by a 3.7-fold change. The negative regulation of endothelial cell proliferation gene, *TNFSF15*, was up-regulated by a 2.5-fold change. The proteolysis gene, *ITGBL1*, the protein amino acid phosphorylation genes, *SGK1* and *RIPK2*, are up-regulated in the WRR chickens. The copper ion transport gene, *SLC31A1*, was localized on chr17:1874758–1884555, and was up-regulated in the WRR chickens by a 7.3-fold change. The *PODXL*, *RAD54B*, *PODXL*, *PMP2* and *TMSB10*, were up-regulated in the WRR chickens. The melanin biosynthesis gene, *DDT*, ion transport genes, *SLC22A2*, and *GLRA1*, lipid metabolism process gene, *P20K* (also known as *EX-FABP*), cellular amino acid metabolism gene, *LOC772201*, protein complex gene, *ATPAF1*, proteolysis genes, *FOLH1*, *C1R*, and *VSP13B*, striated muscle contraction gene, *MYBPC2*, nitrogen compound metabolism process gene, *Vanin1*, porphyrin biosynthesis process gene, *FECH*, and response to stress genes, *HSP70*, *HSP25*, and *HSPB1*, were down-regulated in slow-growing XH chickens. In addition, the signal transduction genes, similar *to KIAA0712* protein, and *ANK2*, small GTPase mediated signal transduction gene, *RAB30*, DNA integration gene, *LOC770705*, amino acid phosphorylation gene, *PRKD3*, carbohydrate metabolic process gene, *CBR1*, and *NAT13*, neuron migration gene, *MDGA1*, hemophilic cell adhesion gene, *PCDH24*, sodium transport gene, *SLC13A5*, regulation of transcript DNA-dependent genes, *CREB3L2*, and *MLL3*, were also down-regulated in slow-growing XH chickens (Table 3).

**Figure 2:**
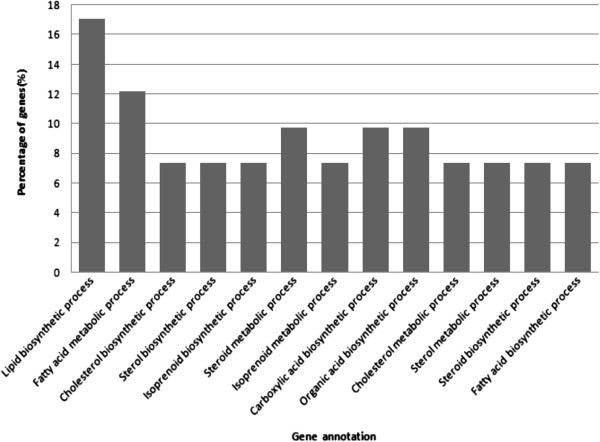
The functional distribution of gene clusters of liver biological process.

Different gene ontology (GO) terms for biological process were identified in the livers of WRR and XH chickens. The highest GO clustered was in lipid biosynthesis process and fatty acid metabolism process (Figure 2).

**Figure 3:**
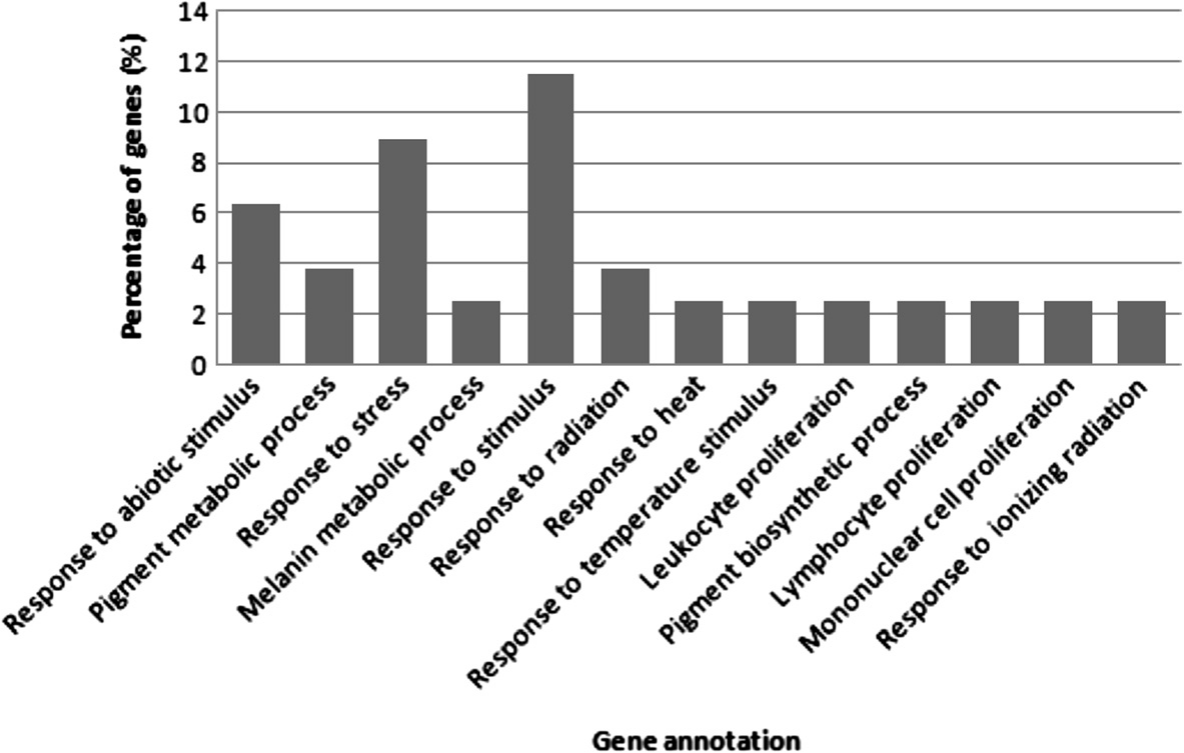
The functional distribution of gene clusters of hypothalamus biological process.

In hypothalamus tissue, the GO terms for biological process in the WRR and XH chickens were mostly observed in response to stimulus, response to stress, and response to abiotic stimulus. Pigment metabolic process, melanin metabolic process, response to radiation, response to heat, response to temperature stimulus, leucocyte proliferation, pigment biosynthesis process, lymphocyte proliferation, mononuclear cell proliferation and response to ionizing radiation were also observed (Figure 3).

In the pathway study, a number of lipid-related genes: A*CSBG2*, *FASN*, *LPL*, *GPAM*, *FDPS*, and others were identified. The cicardian clock gene, *ARNTL* also known as *Bmal1*, was observed.

Based on the pathways, differentially expressed genes participated in several function related to lipid (Tables 2 and 4). The lipid related genes were *ACSBG2*, *SULT1B1*, and *LDLR* of lipid metabolism, *LPL* of glycerolipid metabolism, and *MTTP* of lipid transporter activity, *FASN* and *ELOVL6* of biosynthesis of unsaturated fatty acids, *LSS*, *HMGCR*, *NSDHL*, *DHCR24*, *IDI1*, of *HSD17B7* of biosynthesis of steroid, *AGPAT4* and *FRZB* of triacylglyceride synthesis, *GPAM* of glycerolipid metabolism, *PHOSPHO1* and *PTDSS1* of glycerophospholipid metabolism, *ATP6V1C2* of oxidative phosphorylation, *ACSS2* of glycolysis, *GCNT2* of glycosphingolipid biosynthesis – lactoseries, and *ME1* of pyruvate metabolism (Figure 3).

**Table 4:** Pathways of the fat-deposition-related genes in the liver of WRR and XH chickens

**Probeset ID**	**Gene**	**Pathway**
GgaAffx.12964.1.S1_s_at	*LSS*	Biosynthesis of steroids
Gga.13365.1.S1_at	*AGPAT4*	Triacylglyceride_Synthesis_BiGCaT
GgaAffx.21515.1.S1_s_at	*PTDSS1*	Glycerophospholipid metabolism
GgaAffx.12469.1.S1_at	*ELOVL6*	Biosynthesis of unsaturated fatty acids
Gga.7215.2.S1_a_at	*HSD17B7*	Biosynthesis of steroids
Gga.2334.1.S2_at	*PHOSPHO1*	Glycerophospholipid metabolism
Gga.2298.1.S1_at	*ATP6V1C2*	Oxidative phosphorylation
GgaAffx.5529.1.S1_at	*GPAM*	Glycerolipid metabolism
Gga.8851.1.S1_a_at	*IDI1*	Biosynthesis of steroid
GgaAffx.2094.4.S1_s_at	*ACSS2*	Glycolysis/Gluconeogenesis
Gga.1132.1.S1_at	*ME1*	Pyruvate metabolism
GgaAffx.21769.1.S1_s_at	*LPL*	Glycerolipid metabolism
Gga.9630.1.S1_s_at	*LDLR*	Lipid metabolism
Gga.9949.1.S1_at	*NSDHL*	Biosynthesis of steroids
GgaAffx.12935.1.S1_s_at	*DHCR24*	Biosynthesis of steroids
Gga.2785.1.S1_s_at	*HMGCR*	Biosynthesis of steroids
Gga.2448.1.S2_at	*FASN*	Fatty acid biosynthesis
GgaAffx.8101.1.S1_at	*GCNT2;LOC428479*	Glycosphingolipid biosynthesis - lactoseries
GgaAffx.23852.1.S1_at	*MTTP*	lipid transporter activity
Gga.7792.1.S1_s_at	*ACSBG2*	Lipid metabolism
Gga.8853.2.S1_a_at	*SULT1B1*	Molecular_function--transferase_activity
Gga.4955.1.S1_at	*FRZB*	Adipogenesis; Cellular_component

In hypothalamus tissue, three genes related to VEGF signaling pathway, four genes related to MAPK signaling pathway, one gene each related to alpha-linolenic acid metabolism, nitrogen metabolism, linoleic acid metabolism, porphyrin and chlorophyll metabolism were identified. Then a homologous recombination, heparan sulfate biosynthesis, ether lipid metabolism, arginine and proline metabolism, arachidonic acid metabolism, N-glycan biosynthesis, glycerophospholipid metabolism, ErbB signaling pathway, Wnt signaling pathway were also observed in our present study (Table 5).

**Table 5:** Pathway of the fat-deposition-related genes expressed in hypothalamus tissue of WRR and XH chickens

**Pathway**	**Count**	***P*-Value**	***Q*-Value**	**Gene**
VEGF signaling pathway	3	4,32E-05	2,16E-05	*HSPB1;PLA2G12A;KRAS*
MAPK signaling pathway	4	1,02E-04	3,40E-05	*HSPB1;HSP70;PLA2G12A;KRAS*
alpha-Linolenic acid metabolism	1	0,015	0,001	*PLA2G12A*
Nitrogen metabolism	1	0,019	0,002	*CA3*
Linoleic acid metabolism	1	0,019	0,002	*PLA2G12A*
Porphyrin and chlorophyll metabolism	1	0,023	0,002	*FECH*
Homologous recombination	1	0,026	0,002	*RAD54B*
Heparan sulfate biosynthesis	1	0,026	0,002	*HS6ST2*
Ether lipid metabolism	1	0,030	0,002	*PLA2G12A*
Arginine and proline metabolism	1	0,032	0,002	*LOC396507*
Arachidonic acid metabolism	1	0,033	0,002	*PLA2G12A*
N-Glycan biosynthesis	1	0,043	0,002	*ALG13*
Inositol phosphate metabolism	1	0,052	0,0027	*IPMK*
Glycerophospholipid metabolism	1	0,056	0,002	*PLA2G12A*
ErbB signaling pathway	1	0,086	0,004	*KRAS*
Wnt signaling pathway	1	0,138	0,005	*TCF7L2*

### Validation of differential expression by real-time RT-PCR

The mRNA levels of 9 genes involved in fat deposition were further quantified using real-time RT-PCR (Table 6). The level of *18S rRNA* was chosen as reference and confirmed to be invariable. The expression levels (normalized to 18S) of the 9 genes were determined. Fold changes of gene expression determined by real-time RT-PCR were compared with the fold changes obtained from microarray analysis (Table 6). The highest fold changes in WRR chickens compared with XH chickens were confirmed in the *SULT1B1*, *ACSBG2*, *ELOVL6*, *SLC31A1*, and *PNPLA3* genes. The lowest fold-changes were observed in the *DDT* and *BEAN* genes.

**Table 6:** Comparison of liver tissue gene expression levels between microarray and qRT-PCR

	**Microarray**	**Real-time PCR**
**Genes**	**Fold changes in WRR vs. XH**	**Fold changes in WRR vs. XH**
*SULT1B1*	4,13	3,28
*LPL*	2,5	2,48
*ELOVL6*	2,18	1,76
*ACSBG2*	5,3	2,29
*PNPLA3*	3,03	4,6
*BEAN*	-4,2	-5,76
*SLC31A1*	7,3	1,09
*DDT*	-6,59	-0,4
*PLA2G12A*	-2,8	-2,6

Validation of differentially expressed genes between WRR and XH chickens by RT-PCR.

The data presented indicate the relative mRNA expression of both microarray and qRT-PCR.

Positive values mean that the gene was up-regulated when WRR chickens were compared with XH chickens. Similarly, a negative number means that the gene was down-regulated.

### Expression levels of the Fat deposition related genes in the Fat tissues of WRR and XH chickens

**Figure 4:**
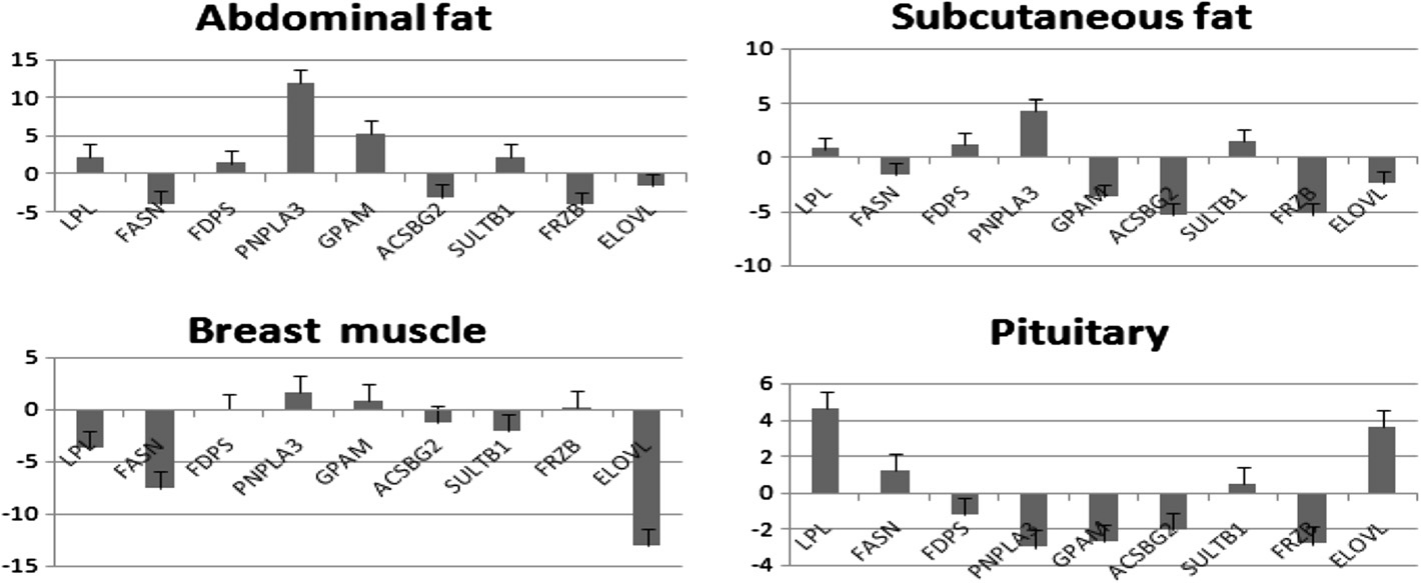
**Data presented indicate the different mRNA gene relative expressions (as fold changes) of FG and SG male chicken relative to different fat tissues.** Error bars represent the standard errors. Positive values imply genes were up-regulated in fast-growing chicken compared to slow-growing ones.

When WRR males were compared with XH males, the expression of the *LPL*, *FDPS*, *PNPLA3*, *GPAM*, and *SULT1B1* genes were up-regulated, and the *FASN*, *ACSBG2*, and *FRZB* were down-regulated in the abdominal fat tissue (Figure 4). In the subcutaneous fat tissue, the *LPL*, *FDPS*, *PNPLA3*, and *SULT1B1* were up-regulated, and the *FASN*, *GPAM*, *ACSBG2*, and *FRZB* genes were down-regulated. In the breast muscle tissues, the *FDPS*, *PNPLA3*, *GPAM*, and *FRZB* were up-regulated, and the *LPL*, *FASN*, *ACSBG2*, *SULT1B1*, and *ELOVL6* genes were down-regulated (Figure 4). In the pituitary tissues, the *LPL*, *FASN*, *SULT1B1*, and *ELOVL6* genes were up-regulated, and the *FDPS*, *PNPLA3*, *GPAM*, *ACSBG2*, and *FRZB* genes were up-regulated (Tables 7, 4 and 5).

**Table 7:** The localization of differentially expressed fat–related genes

**Alignments**	**Gene Symbol**	**Transcript ID**	**Gene ontology**
chrZ:53399697-53408327	*LPL, ENSGALG00000015425*	NM_205282	GO:0004465 lipoprotein lipase activity;
			GO:0004806 triacylglycerol lipase activity;
			GO:0006629 lipid metabolism;
chr4:53309684-53311980	*SULT1B1, SULT1B1*	NM_204545	GO:0006629 lipid metabolism;
			GO:0008202 steroid metabolism;
chr28:1247898-1259038	*ACSBG2(RCJMB04_9i11)*	NM_001012846	006629 lipid metabolism;
			GO:0006631 fatty acid metabolism;
chr15:4477440-4512637	*AACS, ENSGALG00000002899*	NM_001006184	GO:0006629 lipid metabolism;
			GO:0006631 fatty acid metabolism;
			GO:0005829 cytosol
chr1:71256654-71270462	*PNPLA3*	XM_416457	GO:0006629 lipid metabolism;
			GO:0006629 lipid metabolism;
			GO:0016042 lipid catabolism
chr7:2296059-2312305	*FRZB, ENSGALG00000002763*	NM_204772	GO:0017147 Wnt-protein binding;
			GO:0007275 development;
			GO:0016055 Wnt receptor signaling pathway
chr15:8372896-8375331	*DDT(RCJMB04_2c16)*	NM_001030667	GO:0006583 melanin biosynthesis from tyrosine;
chr1:132739051132944192	*DHRSX*	XM_001232713	GO:0055114 oxidation reduction
chr7:6879282-6888069	*LSS*	NM_001006514	GO:0006695 cholesterol biosynthesis
chr7:22713218-22757360	*DPP4*	NM_001031255	GO:0005515 protein binding;
			GO:0008239 dipeptidyl-peptidase activity;
			GO:0042803 protein homodimerization activity;
chr13:13578206-13590970	*MGAT4B*	XM_414605	GO:0005975 carbohydrate metabolism;
chrZ:23472632-23474241	*HMGCR, ENSGALG00000014948*	NM_204485	GO:0004420 hydroxymethylglutaryl-CoA reductase (NADPH) activity;
			GO:0016491 oxidoreductase activity;
			GO:0050661 NADP binding;
chr5:24711230-24725772	*ENSGALG00000008078, FUT8*	NM_001004766	GO:0008424 glycoprotein 6-alpha-L-fucosyltransferase activity;
			GO:0046921 alpha(1,6)-fucosyltransferase activity;
			GO:0007179 transforming growth factor beta receptor signaling pathway
chr6:28077530-28103087	*GPAM*	XM_421757	GO:0006631 fatty acid metabolism;
			GO:0019432 triacylglycerol biosynthesis;
			GO:0040018 positive regulation of body size;
chr20:3629311-3638107	*SGK2*	XM_417346;CR387909	GO:0004713 protein-tyrosine kinase activity;
			GO:0005524 ATP binding;
chr3:34793675-34819927	*PRKD3*	XM_419526	GO:0004713 protein-tyrosine kinase activity;
			GO:0005524 ATP binding;
chr20:2633184-2647420	*ACSS2*	XM_417342	GO:0006085 acetyl-CoA biosynthesis;
Chr1: 64269656-64276867	*LOC418170*	XM_416401	GO:0055114 oxidation reduction
chr6:17648418-17654233	*CYP2C45*	SNM_001001752	GO:0055114 oxidation reduction
chr4:11367346-11377074	*NSDHL*	XM_420279	GO:0005975 carbohydrate metabolism;
			GO:0008203 cholesterol metabolism;
chrUn_random:7545445-7546725	*FDPS*	XM_422855	GO:0006695 cholesterol biosynthesis
chr2:144322566-144334593	*SQLE*	NM_001030953	GO:0055114 oxidation reduction;
chr8:26011324-26019531	*DHCR24*	NM_001031288	GO:0016125 sterol metabolism;
chr1:64293981-64312331	*AKR1B1*	XR_026805	GO:0055114 oxidation reduction
chr1:118069255-119072613	*DMD*	NM_205299	GO:0007519 striated muscle development; cytoplasm;
			GO:0016010 dystrophin-associated glycoprotein complex;
			GO:0045121 lipid raft;
chr18:4906222-4942593	*FASN, ENSGALG00000002747*	NM_205155;J02839	GO:0000036 acyl carrier activity;
			GO:0004312 fatty-acid synthase activity;
			GO:0006633 fatty acid biosynthesis;
chr4:59493262-59560594	*ELOVL6*		GO:0006633 fatty acid biosynthesis
chr6:11960338-11966483	*HKDC1*	XM_421579	GO:0005524 ATP binding;
			GO:0005975 carbohydrate metabolism;
			GO:0006096 glycolysis;
chr2:64406040-64411831	*GCNT2*	XM_426036;XM_418950	GO:0008375 acetylglucosaminyltransferase activity;
chr1:98522850-98669948	*GBE1*	XM_425536	GO:0005975 carbohydrate metabolism

### Polymorphisms of fat deposition genes associated with fat trait in chickens

The SNP rs10731268 of the *ACSBG2* gene was associated with abdominal fat weight (*P* = 0.005), and abdominal fat percentage (*P* = 0.022). The SNP rs15248801 of the *ACSBG2* gene was associated with abdominal fat weight (*P* = 0.039) [Table 8]. The SNP rs15822158 of the *FASN* gene was associated with fat band width (*P* = 0.0003), abdominal fat percentage (*P* = 0.001), and abdominal fat percentage (*P* = 0.005) [Table 9]. The SNP rs15822181 of the *FASN* gene was associated with abdominal fat weight (*P* = 0.049) while the SNP rs16418687 of the *ELOVL6* gene was associated with subcutaneous fat (*P* = 0.034). The SNP rs14092745 of the *DDT* gene was associated with fat band width (*P* = 0.048) [Table 9, 10].

**Table 8:** Association of the G127069A, T1247123C in the ACSBG2 gene with chicken fat traits

**rs10731268 = G1257069A**					**rs15248801 = T1247123C**			
**Traits**	***P*** **value**	**Genotypes**			***P*** **value**	**Genotypes**		
		**GG**	**GA**	**AA**		**TT**	**TC**	**CC**
Fat Bandwidth (mm)	0,587	0	10,05 ± 6,76(33)	13,85 ± 1,80(462)	0,66	11,65 ± 7,34(28)	11,34 ± 3,26(142)	14,75 ± 2,16(325)
Abdominal fat weight (g)	0,005**	0	18,58 ± 2,27(33)b	27,21 ± 0,79(463)a	0,0396*	26,07 ± 3,23(28)	23,68 ± 1,43(142)	28,06 ± 0,94(325)
Abdominal fat percentage	0,3331	0	12,16 ± 9,83(33)	22,02 ± 2,62(463)	0,3627	18,07 ± 57,75(28)	15,61 ± 3,44(142)	21,43 ± 2,27(326)

Means with different letter are significantly different ** (*P* > 0.01); * (*P* > 0.05).

Data are presented at least square means ± SE.

The number shown in parentheses stands for the selected individuals.

**Table 9:** **Association of the G4928024A, C4930169T in the** ***FASN*** **gene with chicken fat traits**

**rs15822158 = G4928024A**					**rs15822181 = C4930169T**			
**Traits**	**P,Value**	**Genotypes**			**P.Value**	**Genotypes**		
		**AA**	**AB**	**BB**		**CC**	**CT**	**TT**
Subcutaneous fat thickness (mm)	0,7051	4,34 ± 0,39(14)	3,94 ± 0,29(26)	4,03 ± 0,07(438)	0,8198	4,02 ± 0,07(352)	4,12 ± 0,14(107)	4,03 ± 0,33(20)
Fat Bandwidth (mm)	0,0003^**^	10,52 ± 10,4(14)b	44,02 ± 7,6(26)a	11,98 ± 1,86(437)b	0,8934	14,21 ± 2,11(351)	12,32 ± 3,82(107)	11,96 ± 8,84(20)
Abdominal fat weight (g)	0,2155	21,98 ± 4,60(14)	22,47 ± 3,37(26)	27,28 ± 0,82(437)	0,0491*	25,97 ± 0,92(351)	30,54 ± 1,66(107)	24,85 ± 3,85(20)
Abdominal fat percentage	0,0016**	15,52 ± 11,02(14)b	48,12 ± 0,09(26)a	18,18 ± 1,97(438)b	0,9755	19,71 ± 2,22(352)	20,34 ± 4,04(107)	18,18 ± 9,34(20)

Means with different letter are significantly different ** (*P* > 0, 01); * (*P* > 0,05).

Data are presented at least square means ± SE.

The number shown in parentheses stands for the selected individuals.

**Table 10:** **Association of the A59539099G in the** ***ELOVL 6*** **and the A8378815G in the** ***DDT*** **gene on chicken fat traits**

**chr4/ELOVL 6**					**chr, 15/DDT**			
**rs16418687 = A59539099G**					**rs14092745 = A8378815G**			
**Traits**	***P*** **value**	**Genotypes**			***P*** **value**	**Genotypes**		
		**AA**	**AG**	**GG**		**AA**	**AG**	**GG**
Subcutaneous fat thickness (mm)	0,033*	4,05 ± 0,06(475)a	1,820 ± 1,04(2)	0	0,100	4,31 ± 0,18(60)	4,08 ± 0,09(229)	3,96 ± 0,11(185)
Fat Bandwidth (mm)	0,867	16,34 ± 3,19(475)	8,08 ± 49,23(2)	0	0,048*	27,04 ± 5,07(60)	12,02 ± 2,60(228)	11,55 ± 2,89(185)
Abdominal fat weight (g)	0,338	26,84 ± 0,79(474)	15,15 ± 12,16(2)	0	0,649	28,18 ± 5,54(60)	31,17 ± 2,83(229)	26,33 ± 3,16(185)
Abdominal fat percentage	0,811	23,42 ± 3,22(475)	11,47 ± 49,75(2)	0	0,254	34,17 ± 7,41(60)	22,17 ± 3,79(229)	17,25 ± 4,22(185)

Means with different letter are significantly different ** (*P* > 0.01); * (*P* > 0.05).

Data are presented at least square means ± SE.

The number shown in parentheses stands for the selected individuals.

## Discussion

The approach of selective-fat-deposition-related-genes in animals is a relatively new strategy aimed at improving production efficiency while enhancing meat quality. Efforts to reduce fat deposition in animals include genetic selection, feeding strategies, housing and environmental strategies as well as hormone supplementation. While these efforts have improved production efficiency and reduced carcass lipid deposition, negatively impacts on meat quality were due to reduced intramuscular fat deposition [22]. Based on the comparison of two types of breeds of chicken whose fat deposition and growth rate are exceptionally varied, a functional genomics approach was chosen in order to identify chicken fat-deposition-related-genes. In this genomic approach, liver tissue was used. The liver is the site of fat synthesis, and hypothalamus, which is a major gland for the endocrine system. Few studies focused on global gene expression surveys in chickens. Wang *et al.*[19] provided analysis of chicken adipose tissue gene expression profile. Other hepatic transcriptional analyses had been reported, using dedicated chicken 3.2 K liver-specific microarray [14,23] or a 323 cDNA microarray [24].

Differential gene expressions in liver during the fat developmental stage in fast growing WRR and slow growing XH chickens were related to lipid metabolism in our study. It has been reported that some genes, e.g. *3-hydroxyacyl-CoA dehydrogenase*, *long chain acyl-CoA thioesterase*, *fatty-acid elongation enzymes* and *cytosolic fatty-acid- and acyl-CoA-binding proteins*, are known to play key roles in mammalian fat or lipid metabolism [25]. Glyco-metabolism such as glycol-sphingolipids (*GCNT2*), biosynthesis of steroids, fatty acid biosynthesis (*ELOVL6* and *FASN*) was observed in this study. Collin *et al.*[26] reported that fast growing chickens developed excessive adiposity besides the high muscle mass resulting from selection. The suggestion is that differential expression of the lipid metabolism related genes might be one of the factors in the differences of fat deposition between fast growing and slow growing chickens at the developmental stage.

The liver is the main site for fatty acid biosynthesis and the fatty acids are then transported to the adipose tissue for storage. The tasks are accomplished through the generation of triglycerides by the liver from fatty acids and L-a-glycerophosphate, packaged into very low density lipoproteins (VLDL), and then, secreted into the blood*.* The triglycerides in VLDL are processed by the adipose tissue and finally deposited in the central vacuole of the adipocyte. It was suggested that several mechanisms regulate intracellular non-esterified fatty acids composition, including fatty acid transport, acyl CoA synthetases, fatty acid elongases, desaturases, neutral and polar lipid lipases and fatty acid oxidation. Most of these mechanisms are regulated by PPAR alpha or SREBP-1c. Together, these mechanisms control hepatic lipid composition and affect whole-body lipid composition [27]. LPL catalyzes the hydrolysis of plasma lipoproteins, which is a rate-limiting step in the transportation of lipids into peripheral tissues [28,29]. The *LPL* gene expression in fast growing chicken was 2.5-fold greater than that in the slow growing type at the developmental stage in this study. In mammals, increased LPL activity is strongly associated with fat deposition and obesity, and these are regulated by both insulin and glucocorticoids according to Fried *et al.*[30]. The major site of lipogenesis in birds, however, is the liver rather than the adipose tissue [31]. The role of fatty acid-binding protein in the intramuscular trafficking of long-chain fatty acids within intramuscular adipocytes has been studied and found to be related to intramuscular levels in different species [32,33].

Fatty acid synthesis (FAS) occurs during periods of energy surplus and concomitantly its gene expression is down-regulated during starvation in the liver [34], which is the major site of lipogenesis in avian species [35-37]. The regulation of hypothalamic fatty acid synthesis gene expression in response to starvation is similar to that of liver fatty acid synthesis. In birds, like in humans, fatty acid synthesis primarily occurs in the liver. Demeure *et al.*[38] reported that chicken *FASN* gene is directly the target of liver cross receptor (LxR) alpha and therefore, expands the role of LxR alpha as a regulator of lipid metabolism. *FASN* and *GPAM* are two enzymes that play central roles in *de novo* lipogenesis. The G4928024A of the *FASN* gene is significantly associated with fat band width, abdominal fat percentage, and abdominal fat percentage.

The *DDT* gene was observed down-regulated in both tissues when fast growing WRR chickens were compared with slow growing XH chickens. This gene has function in melanization which can play a role in the pigmentation of abdominal fat. It also, has a high correlation with the accumulation of melanin in the skin of the shanks. Melanization of abdominal fascia is not harmful but it may cause severe economic losses to the producer. It was surprising to observe that the *FDPS*, *LSS*, *HMGCR*, *NSDHL*, *DHCR24*, *IDI1*, and *HSD17B7* were up-regulated in fast growing WRR chickens. These genes are considered as the ones which has some functions in cholesterol biosynthesis. The glycolytic genes (*ACSS2*), carbohydrate metabolic and fatty acid biosynthesis were also up-regulated in the WRR chickens. It is suggested that the genes related to cholesterol biosynthesis, carbohydrate metabolic and fatty acid biosynthesis may have influence on fat development.

This study also showed that the genes related to proline biosynthetic process, *member 2* of *pyrroline-5-carboxylate reductase* family, *member A1* of *aldehyde dehydrogenase 18* families, and oxidation reduction, *CYP1A4*, *CYP1A1*, *AKR1B1*, *CYP4V2*, *DDO*, and *similar to aldose reductase*, were differently expressed between the WRR and XH chickens. The *CYP2H1*, *CYP2C45*, *CYP2C18*, *MICAL1* and *CYP3A37* genes were significantly different in between the WRR and XH chickens. In this study, many lipid-related genes were identified, *ACSBG2*, *FASN*, *LPL*, *GPAM*, and *FDPS*. The cicardian clock gene (*ARNTL)* was observed, it plays a role in glucose, lipid metabolism and adipogenesis [39-41]. Moreover, a network of 11 genes, *LPL*, *ACSBG2*, *AACS*, *FASN*, *LSS*, *FDPS*, *SULT1B1*, *HMGCR*, *DPP4*, *FUT8*, and *PLAU*, was observed. Parallel expression patterns of these functionally relevant genes provided strong evidence for their coordinated involvement in lipid biosynthesis, cholesterol biosynthesis and fatty acid degradation in chickens. In chickens, the *ACSBG2* gene has been found to play a significant role in lipid metabolism. The present study confirmed this conclusion.

In order to support the results of the microarray study, all the genes used for the mRNA assay were found to have good relationship with fat-related genes as their functions related to lipid metabolism, cholesterol biosynthesis and fatty acid metabolism. Interestingly, the *SULT1B1*, *PNPLA3*, *GPAM*, *ELOVL6*, *LPL*, *FASN*, *ACSBG2*, *FDPS*, and *FRZB* genes were preferentially expressed in 4 fatty tissues of abdominal fat, subcutaneous fat, breast muscle and pituitary gland when WRR were compared with XH chickens. However, *PNPLA3* mRNA level was higher in all tissues in WRR except pituitary tissue, where it expressed lower levels in XH chicken. *PNPLA3*, also referred to as adiponutrin, was originally identified as a highly adipose–specific transcript that rapidly responds to nutritional status [42]. The microarray assay demonstrated that there was a 3 times higher expression of the *PNPLA3* gene in liver tissue at 8 wk of age in WRR than in XH chickens. It could be concluded that the *PNPLA3* gene is involved in fat deposition.

The microarray data showed that the *SULT1B1* is abundantly expressed in liver tissue with 7 fold change in WRR with XH chickens. The gene was reported to be expressed in liver and other numerous extra-hepatic tissues [43]. *FDPS* is an important intermediate in cholesterol and sterol biosynthesis, a substrate for protein farnesylation and geranylgeranylation, and a ligand or agonist for certain hormone receptors and growth receptors. In this study, the *FDPS* was found to belong to the cholesterol biosythetic group. The *FDPS* mRNA level was higher in subcutaneous fat and pituitary tissue of WRR female chicken against XH counterpart.

The *GPAM* gene plays a vital role in the regulation of cellular triacylglycerol and phospholipid levels [44,45]. In this study, adipose tissues such as abdominal fat and subcutaneous fat were found to have the highest levels of *GPAM* mRNA expression whereas it was rarely detectable in the liver in the microarray assay. The *FRZB* gene (also known as *SFRP3*) is a member of the secreted frizzled receptor family of soluble proteins which binds to and antagonises Wnt receptor [46]. Wnts are secreted lipid-modified signaling proteins that influence multiple processes in the development of animals. The *FRZB* was shown to play a major role in adipogenesis in the microarray analysis of WRR and XH at 8 wk of age. *ELOVL6* is involved in *de novo* lipogenesis and is regulated by dietary, hormonal and developmental factors [47]. In this study, *ELOVL6* mRNA level was higher in all tissues of XH chickens than of WRR chickens.

LPL is a glycoprotein enzyme that is produced in several tissues of mammals such as adipose tissue, skeletal muscle, heart, macrophages and lactating mammary gland, but not in the liver of adults [48,49]. In chickens, LPL hydrolyzes lipids in lipoproteins, such as those found in chylomicrons and very low-density lipoproteins (VLDL) into three free fatty acid molecules and one glycerol molecule [29,50-52]. In studying the deposition of fat in the abdominal fat pads of chicken, it has become clearer that LPL-catalyzed hydrolysis of triacylglycerol in adipose tissue is a rate-limiting step in fat accumulation [28]. Therefore, the transport and incorporation of exogenous lipids, i.e. plasma VLDL lipoprotein and portomicron, are essential for the deposition of cytoplasmic triglycerides in abdominal adipose tissue. These are characteristics of lipid metabolism in avian species since lipogenic activity is much greater in the liver than in adipose tissue [28,53,54]. This study showed that the *LPL* gene expression was significantly higher in fast-growing chickens than in slow-growing chickens.

The association study provides direct evidence of genes related to fat deposition. In our association study, the A59539099G of the *ELOVL6* gene was significantly associated with subcutaneous fat. The A8378815G of the *DDT* gene was associated with fat band width. The C4930169T of the *FASN* gene was also found to be associated with abdominal fat weight. G1257069A and T1247123C of the *ACSBG2* gene were significantly associated with fat traits. The above results further confirmed that the *ELOVL6*, *DDT*, *FASN*, and *ACSBG2* genes are related to chicken fat deposition.

## Conclusion

The differential genes expressions in fast and slow growing chickens show differences in fat developmental stage which is supported by lipid-related genes identified and characterized in these two types of chicken. The findings indicate that the variation of the *ACSBG2*, *FASN*, *ELOVL 6*, and *DDT* genes were significantly associated with fat deposition.

## Abbreviations

WRR: White recessive rock; XH: Xinghua; RMA: Robust multi-array; MAS: Molecule annotation system; KEGG: Kyoto encyclopedia of genes and genomes; AgriGO: GO analysis toolkit and database for agricultural community; DAVID: Database for annotation, visualization and integrated discovery.

## Competing interests

The authors declare that they have no competing interests.

## Authors’ contributions

HCA is a correspondence author, conducted all the experiments and written and approved the final manuscript. WP participated in data analysis and approved the final manuscript. SX participated in data collection, laboratory experiment and approved final manuscript. JX participated in data collection, laboratory experiment and approved final manuscript. ZR participated in data collection, laboratory experiment and approved final manuscript. SL corried out the data analysis and approved final manuscript. ZX guided in gene expression analysis and approved final manuscript.
